# Changes in oral health-related quality of life after treatment of hypersensitive molar incisor hypomineralization–affected molars with a sealing

**DOI:** 10.1007/s00784-021-03947-z

**Published:** 2021-04-20

**Authors:** Katrin Bekes, Stefanie Amend, Julia Priller, Claudia Zamek, Tanja Stamm, Norbert Krämer

**Affiliations:** 1 Department of Paediatric Dentistry, Medical University of Vienna, University Clinic of Dentistry, Sensengasse 2a, 1090 Vienna, Austria; 2 Department of Paediatric Dentistry, Medical Centre for Dentistry Section for Outcomes Research, University Medical Center Giessen and Marburg, Campus Giessen, Schlangenzahl 14, 35392 Giessen, Germany; 3 Private Practice, Graf-Adolf-Straße 24, 40212 Düsseldorf, Germany; 4 Center for Medical Statistics, Informatics, and Intelligent Systems, Section for Outcomes Research, Medical University of Vienna, Spitalgasse 23, 1090 Vienna, Austria

**Keywords:** Molar incisor hypomineralization (MIH), Hypersensitivity, Oral health-related quality of life (OHRQoL), Child Perceptions Questionnaire (CPQ)

## Abstract

**Objectives:**

The aim of this study was to investigate the changes in oral health-related quality of life (OHRQoL) before and after treatment of hypersensitive molars affected by molar incisor hypomineralization (MIH) with a sealing.

**Methods:**

Thirty-eight children with two MIH-affected molars showing hypersensitivity and non-occlusal breakdowns were included. Hypersensitivity was assessed with an evaporative (air) stimulus. Two affected teeth were sealed by two calibrated operators using a split-mouth design: Clinpro Sealant in combination with Scotchbond Universal, and Ketac Universal (3M), respectively. OHRQoL was measured using the German version of the CPQ8–10 (CPQ-G8–10) at baseline, and after 1, 4, 8, and 12 weeks, respectively.

**Results:**

The CPQ total score decreased significantly from a mean of 14.7 (±5.9) to 6.4 (±4.7) (*p* < 0.001) 1 week after treatment revealing improved OHRQoL. After 12 weeks, OHRQoL improved again proven by a decreased mean score of 2.7 (±3.2).

**Conclusions:**

Sealing of hypersensitive MIH-affected molars revealed a significant improvement of OHRQoL immediately and throughout the 12-week follow-up.

**Clinical relevance:**

Hypersensitivity can be a major complaint in patients with MIH. This is the first study evaluating the effect of sealing on OHRQoL in affected patients.

## Introduction

Since its first introduction in 2001, there has been an increasing interest in molar incisor hypomineralization (MIH). The term is defined as enamel hypomineralization of systemic origin affecting one or more first permanent molars that are associated frequently with affected incisors [1]. Although MIH is considered to be an idiopathic condition, its concise etiology remains unclear [2]. Patients affected by MIH can present several clinical problems. Depending on the severity, MIH teeth can show rapid wear, enamel loss, increased susceptibility to caries, loss of fillings, and most of all, severe hypersensitivity often resulting in severe discomfort [3]. With respect to hypersensitivity, children often report that hot and cold or sweet drinks and meals, toothbrushing, and even air flow cause pain [1, 4, 5]. The underlying mechanism is not fully understood, but it is believed that the high porosity of the affected enamel favors the penetration of bacteria in the dentinal tubules, causing a subclinical pulpal inflammation [6].

Nowadays, the concept of oral health-related quality of life (OHRQoL) has become important to assess oral health status in children and in adults [7] as clinical indicators alone do not reveal the full impact of oral conditions on the psychosocial well-being of a patient [8]. The subjective evaluation of OHRQoL “reflects people’s comfort when eating, sleeping and engaging in social interaction; their self-esteem; and their satisfaction with respect to their oral health” [9]. For children aged 8 to 10 years, the Child Perceptions Questionnaire (CPQ8–10) is the most frequently used instrument to measure OHRQoL in this age group [10]. This generic questionnaire was designed to cover a variety of oral conditions.

Although MIH is known for many years now, there is still scarce data about the relationship between MIH and OHRQoL [11–13]. Moreover, no study is available to determine the value of hypersensitivity treatment in these patients. To date, only one study has focused on the children’s perspectives with regard to aesthetic interventions in MIH-affected incisors using a validated OHRQoL questionnaire [14].

Therefore, the aim of our study was to investigate the changes in OHRQoL before, and at different time points after treatment of hypersensitive molars affected by MIH using the CPQ questionnaire.

The hypothesis tested in the present paper was that sealing of hypersensitive MIH molars increased OHRQoL in affected patients.

## Materials and methods

### Subjects and setting

For this prospective multi-center study, Austrian and German children aged 6 to 10 years were recruited from Department for Paediatric Dentistry of the University Clinic of Dentistry in Vienna, Austria, and a private practice in Duesseldorf, Germany. The criteria proposed by the European Academy of Paediatric Dentistry (EAPD) [15] were used for the diagnosis of MIH. These include the presence of demarcated opacities, posteruptive enamel breakdown, atypical restorations, and extraction due to MIH in at least one first permanent molar. Demarcated opacities with a diameter of < 1 mm were not considered in the analysis.

German-speaking children with two MIH-affected molars showing hypersensitivity (Schiff Cold Air Sensitivity Scale (SCASS) 2 or 3 [16]) and non-occlusal breakdowns (MIH-TNI 3) [17, 18] were included. Exclusion criteria were systemic diseases, long-term medication, hypomineralized molars due to other medical conditions, hypersensitive study teeth with contributing aetiologies other than recognized clinically as being associated with MIH, caries, and restorations in study teeth.

One proficient and calibrated dentist at each study center examined potential children for inclusion into the trial. Possible MIH-affected molars for inclusion were selected in response to an air blast stimulus. The air was delivered from a standard dental unit air syringe for 1 s at a distance of 1 cm and perpendicular to the occlusal surface of the tooth. Neighboring teeth were shielded with cotton rolls or with the fingers of the examiner. The SCASS was used to assess subject response to this stimulus (0 = subject does not respond to the stimulus; 1 = subject does not respond to the stimulus, but considers stimulus to be painful; 2 = subject responds to air stimulus and moves from the stimulus; 3 = subject responds to air stimulus, moves from the stimulus, and requests immediate discontinuation of the stimulus) [16].

Both included MIH teeth were sealed by one calibrated dentist at each study center using a split-mouth design: Clinpro Sealant in combination with Scotchbond Universal, and Ketac Universal (3M, Seefeld, Germany), respectively. Clinpro Sealant is a light-cure, resin-based fluoride releasing pit and fissure sealant, and Scotchbond Universal is a one-component, light-curing adhesive; Ketac Universal is a radiopaque glass ionomer cement. Before sealing, the tooth surface was cleaned with Clinpro Prophy Paste (3M, Seefeld, Germany) and a bristle brush in order to remove adherent plaque and debris. In both cases, isolation was performed using cotton rolls and a four-hand technique. On one tooth, Scotchbond Universal was applied and rubbed in for 20 s. Then, the adhesive was air dried for approximately 5 s to evaporate the solvent, followed by the application of the light-cure fissure sealant Clinpro Sealant using a syringe. Finally, the sealing was light-cured. If air bubbles were present, these were teased out of the material before curing the sealant. On the other tooth, Ketac Universal was applied. After activating and mixing the capsule according to the manufacturer’s instructions and starting the chemical reaction, the cement was applied to the cleaned fissure. Afterwards, it was covered with Ketac Bond (3M, Seefeld, Germany) and cured. Occlusion control was performed in both sealed teeth using articulating paper. If necessary, adjustments with finishing burs were made. For the duration of the study, patients were advised to use the toothpaste given to them (Clinpro Tooth Crème, 3M; 0.21% sodium fluoride (950 ppm) and functionalized tri-calcium phosphate ingredient (fTCP)).

Randomization was performed with Excel (Microsoft, Redmond, USA). Clinpro Sealant represented treatment A; Ketac represented treatment B. The two teeth that were chosen for the sealing were sorted by their quadrants.

The enrollment into this study was voluntary. Extended information leaflets on the aim of the study were handed out and explained to the parents who gave their written and oral informed consent. The approval for the study procedures was granted by the ethics committee of the local University Review Board (Medical University of Vienna, #1091-2017; University of Giessen, AZ 212/17).

Sample size calculation was performed for the primary endpoint of the study project which focused on the effect of treatment regarding the reduction of hypersensitivity after 12 weeks using two different materials. Measurement of OHRQoL, which is the focus of the present paper, was determined to be the second endpoint of the study. Based on previous studies, a reduction of hypersensitivity by two points on the scale of 0–3 was assumed to be clinically relevant. With a required power of 95% and a significance of 5%, 47 individuals were required. The plan was to conservatively enroll at least 52 participants.

### Data collection

To assess the child’s OHRQoL before and at different time points after treatment, the validated German version of the CPQ8–10 was used [19]. The CPQ8–10 contains a total of 25 items which can be subdivided into four domains: oral symptoms (five items), functional limitations (five items), emotional well-being (five items), and social well-being (ten items). Questions ask about the frequency of events in the child’s last 4 weeks. Responses are made on an ordinal scale (0 = never, 1 = once/twice, 2 = sometimes, 3 = often, 4 = every day/almost every day). Higher scores refer to a worse OHRQoL status. Summing the response codes for the questionnaire items generates domain scores/sub-scales and an overall CPQ-G8–10 score. The instrument’s summary score ranges from 0 to 100. A summary score of zero indicates the absence of any problems, and higher CPQ scores represent more impaired OHRQoL. In addition to the 25 items, the CPQ8–10 includes two questions asking the child for a global rating of the oral health and the overall well-being. These global ratings had a five-point response format (excellent, very good, good, moderate, poor).

The treated teeth were evaluated immediately after treatment and after 1, 4, 8, and 12 weeks and their pain sensitivity is measured again. A photographic documentation was made at the last follow-up examination.

### Data analysis

We tested SCASS and OHRQoL scores for normal distribution using histograms and Shapiro tests. We chose parameter-free tests for non-normally distributed data. OHRQoL scores were compared in a pairwise analysis between two time points each using a *t*-test or a Wilcoxon signed-rank test, depending on the distribution of the data. We corrected the significance level for multiple testing on the basis of the Bonferroni-Holm procedure. The analysis was performed using the statistical program SPSS 26.0 (IBM, Chicago, USA).

## Results

Thirty-nine patients were recruited over 2 years. One child was excluded because the follow-up CPQs contained more missing items than allowed. Therefore, a total of thirty-eight patients with mean age of 7.5 years (SD = 1.6, age range from 6 to 10 years) were included in the analysis. Of these, 18 (47.4 %) were girls. The patients showed a mean dmft of 0.39 (±1.46) and a mean DMFT of 0.21 (±1.46), respectively (Table 1).

**Table 1:** General characteristics of participants

Patient variable	*n* (%), mean (SD; range)
Age (years)	7.5 (1.6, 6–10)
Gender	
Male	20 (52.6)
Female	18 (47.4)
Caries index	
dmft	0.39 (±1.46; 0–8)
DMFT	0.21 (±0.41; 0–1)
Hypersensitivity	
SCASS	2.4 (0.8; 2–3)
VAS	7.0 (1.9; 2–10)

Before treatment, all included patients showed two MIH molars with severe hypersensitivity (Schiff score 2.4 [±0.8; range = 2–3]; VAS score 7.0 [±1.9; range = 2–10]). The mean CPQ-G8–10 score at baseline (before treatment) was 14.7 (±5.9; range = 4–31) (Table 2). The score decreased significantly following treatment at the 1-week follow-up to 6.4 (±4.7; range = 0–17) (*p*<0.001, *t*-test) showing a large effect size (Table 3). The score was significantly reduced again after the 12-week follow-up (±2.7 [3.2; range = 0–14]) (*p*<0.001, *t*-test), also showing a large effect size. This suggested that children perceived themselves as having a marked improvement in their overall OHRQoL. The same results were also observed in all subdomains (Table 2). The scores of the subdomains of the CPQ-G8–10 also decreased significantly (*p* < 0.001, *t*-test) between baseline and the first follow-up presenting an improvement in OHRQoL. A further reduction was seen after 12 weeks in all subdomains (*p*<0.001, *t*-test). As expected, the patients showed high scores in the subdomain “oral symptoms” and “functional limitations.” These include questions on the frequency of pain and the frequency of food intake restriction.

**Table 2:** Mean CPQ scores before treatment at baseline and at follow-ups after 1, 4, 8, and 12 weeks after treatment

*N* = 38	CPQ sum	Oral symptoms	Functional limitations	Emotional well-being	Social well-being
Baseline	14.7 (±5.9)	7.5 (±3.0)	4.5 (±1.6)	1.8 (±1.9)	0.9 (±1.9)
1 week post	6.4 (±4.7)	3.2 (±2.3)	1.8 (±2.2)	0.9 (±1.2)	0.5 (±1.1)
4 weeks post	3.6 (±3.2)	2.1 (±1.8)	0.9 (±1.3)	0.4 (±0.8)	0.2 (±0.6)
8 weeks post	3.0 (±2.7)	2.0 (±1.9)	0.6 (±1.0)	0.2 (±0.7)	0.2 (±0.7)
12 weeks post	2.7 (±3.2)	1.6 (±1.9)	0.6 (±1.1)	0.2 (±0.6)	0.3 (±0.6)

**Table 3:** Change in CPQ sums and effect sizes at different time points

CPQ sum	*p*-value	Change score Mean (SD)	Effect size	Description
Baseline				
– 1 week post	<0.001	8.3 (±7.5)	1.1	Large
– 4 weeks post	<0.001	11.1 (±6.9)	1.6	Large
– 8 weeks post	<0.001	11.7 (±6.2)	1.9	Large
– 12 weeks post	<0.001	12.1 (±6.4)	1.9	Large
1 week post				
– 4 weeks post	=0.001	2.8 (±4.9)	0.6	Moderate
– 8 weeks post	<0.001	3.4 (±4.2)	0.8	Large
– 12 weeks post	<0.001	3.8 (±4.8)	0.8	Large
4 weeks post				
– 8 weeks post	=0.367	0.6 (±3.7)	0.2	Small
– 12 weeks post	=0.201	0.9 (±4.4)	0.2	Small
8 weeks post				
– 12 weeks post	=0.396	0.4 (±2.6)	0.2	Small

## Discussion

To the best of our knowledge, this is the first study evaluating the effect of hypersensitivity treatment of MIH-affected molars on OHRQoL worldwide. Although some authors have already studied the effect of MIH on OHRQoL [11–14, 20–22], intervention studies considering the patient’s view in terms of treatment of molars are lacking. For the first time, this study shows that treatment of hypersensitive molars with a sealing technique leads to positive changes in the child’s quality of life, which is evident by the significant decrease in the overall CPQ scores. Significant improvements in OHRQoL of life were already observed 1 week after treatment in these patients suffering from hypersensitive MIH molars. This development was stable over 12 weeks—supporting the created hypothesis.

In the present study, the CPQ8–10 was used to measure OHRQoL in 6–10-year-old children. The instrument is frequently used worldwide and has already been translated and validated in several countries. In our study, the German version was applied [19]. Originally, the CPQ8–10 was developed for children aged 8–10 years. We extended this age range in our study, including patients from the age of six. However, this is not an unusual step. Other studies focusing on OHRQoL in similar age groups also applied this instrument. For example, Dias et al. evaluated the impact of MIH on OHRQoL in a sample of 6–10-year-old Brazil children using the same questionnaire [12]. It is known that around the age of 6 marks the beginning of abstract thinking and self-concept for children. Children in this age group start to compare their physical features and personality traits, either with those of other children or against a norm [23].

Before treatment, included MIH patients showed a mean CPQ-G8–10 score of 14.7 (±5.9). This is in agreement with other studies. Velandia et al. assessed the influence of MIH on OHRQoL in Columbian children aged 7 to 10 years. The average CPQ8–10 score for participants with MIH was similar (17.4 [±5.9]) [21]. A recent study done in Brazil on a sample of 6–12-year-old schoolchildren using the CPQ8–10 and the CPQ11–14 confirmed the negative impact of MIH on OHRQoL [12]. Mean CPQ scores in children up to 10 years were 15.11 (±10.93).

As this is the first clinical trial to examine OHRQoL before and after hypersensitivity treatment of MIH molars at different time points, no direct comparisons can be made between the current and previous data. Currently, there is only one study studying the effect of treatment in MIH patients. Hasmun et al. (2018) examined 7–16-year-old children before and 1 month after treatment of MIH-affected incisors by reducing the visibility of enamel opacities using the Child Oral Health Impact Profile (COHIP) [14]. The authors were able to show that minimally invasive dental treatment in MIH can have a positive impact on children’s well-being.

To our knowledge, only two studies exist focusing on hypersensitivity treatment in molars [24, 25]. Bekes et al. evaluated the effect of desensitizing agents containing 8% arginine and calcium carbonate for hypersensitivity relief in MIH-affected molars in an 8-week clinical study [24]. Nineteen children received a single in-office treatment with a desensitizing paste containing 8% arginine and calcium carbonate, followed by 8 weeks of brushing twice daily with a desensitizing toothpaste and using the corresponding mouthwash. Application of the desensitizing paste decreased hypersensitivity significantly immediately and throughout the 8-week recalls. Pasini et al. compared the sensitivity of teeth with MIH in children before and after the use of casein phosphopeptide and amorphous calcium phosphate (CPP-ACP) or fluoride toothpaste [25]. After 120 days, the use of the remineralizing agent containing CPP-ACP resulted in a significant improvement in dental sensitivity in patients with MIH.

This study was able to show that sealing of hypersensitive MIH molars with either a composite material or a glass ionomer cement was able to improve OHRQoL in our patients immediately. However, it is not possible to compare the effect of both materials on OHRQoL as every patient got the sealing in a split-mouth design. The clinical effect on hypersensitivity relief regarding the sealings separately will be discussed elsewhere. This paper focuses on the effect of a sealing on OHRQoL in general. It was found that the CPQ score increased significantly following treatment at the 1-week follow-up and was significantly reduced again after the 12-week follow-up. This shows that children perceived themselves as having a marked improvement in their overall OHRQoL. The same results were also observed in all subdomains. The biggest improvements were seen in the oral symptoms domain (7.5 [±3.0] to 3.2 [±2.3]) as well as the functional limitations (4.5 [±1.6] to 1.8 [±2.2]), respectively. These findings were also supported by reaching the minimally important difference (MID) of the CPQ. MID is defined as smallest difference in score which patients perceive as beneficial and which would mandate, in the absence of troublesome side effects and excessive cost, a change in the patient’s management [26]. Mean score change in the total CPQ8–10 score was higher than the MID calculated by Martins-Júnior et al. [27].

One of the major limitations of this study is that the projected sample size was not reached. Due to rigorous inclusion criteria that allowed only hypersensitive MIH molars with no sign of posteruptive breakdown at any surface being included, both study centers were only able to include 39 patients over the large period of 2 years. Therefore, the study was terminated before reaching the targeted sample size. This might have affected the interpretation of our results. However, all included patients showed similar results regarding the significant increase in self-perceived OHRQoL. We assume that the missing patients would have experienced improvements likewise. Another limitation of this study is the lack of a negative control (group without sealing), which also might have influenced the interpretation of our results. Nevertheless, we considered it unethical to have a negative control. Other limitations include the subjective nature of hypersensitivity assessment and the knowledge of participating in a trial. However, compliance bias could not have influenced participant responses as they were not personally related to the investigator nor were they offered any incentive to participate in the trial.

## Conclusion

Sealing of hypersensitive MIH-affected molars with either a composite material or a glass ionomer cement revealed a significant improvement of OHRQoL immediately and throughout the 12-week follow-up.
